# The impact of parental overprotection on the emotions and behaviors of pediatric hematologic cancer patients: a multicenter cross-sectional study

**DOI:** 10.3389/fpsyg.2023.1290608

**Published:** 2024-01-16

**Authors:** Yahui Yu, Xiaofeng Zheng, Wenjing Xu, Yuru Huang, Xulu Wang, Wanting Hong, Runping Wang, Xiaojing Ye, Chunmei Zhang

**Affiliations:** ^1^The Second Affiliated Hospital of Wenzhou Medical University, Wenzhou, Zhejiang, China; ^2^Department of Hematology, The Second Affiliated Hospital of Wenzhou Medical University, Wenzhou, Zhejiang, China; ^3^School of Nursing, Wenzhou Medical University, Wenzhou, Zhejiang, China

**Keywords:** emotional and behavioral problems, blood cancer, parental overprotection, children, cross-sectional studies

## Abstract

**Background:**

Parental overprotection may have an impact on children’s emotional and behavioral problems (EBPs). As pediatric hematologic cancer patients have compromised immune systems, parents of such children often worry excessively, interfering with their daily lives. Therefore, avoiding overprotection is crucial for the overall physical and mental health of pediatric hematologic cancer patients.

**Aims:**

The aim of this study was to examine the current status of EBPs in pediatric hematologic cancer patients and analyze their associated risk factors.

**Design:**

This work was a multicenter cross-sectional observational and correlational study. We collected data anonymously through parental questionnaires from three pediatric hematologic oncology hospitals in China. The Strengths and Difficulties Questionnaire, the Parental Overprotection Measure (POM) scale, and a general information survey designed by the research team were employed to assess children’s EBPs, the degree of parental overprotection, as well as basic demographic and disease-related information. Chi-square tests and generalized linear mixed-effects regression analysis were used to analyze the factors influencing EBPs among the pediatric hematologic cancer patients.

**Setting and participants:**

Using a convenience sampling method, a total of 202 participants’ parents were selected. All participants were invited to complete the questionnaire through one-on-one guidance.

**Results:**

Emotional symptoms accounted for the highest proportion of abnormal EBPs in children (27.72%), followed by peer problems (26.24%), prosocial behavior (25.74%), behavioral problems (14.36%), and total difficulties (13.86%). A minority of children had abnormal hyperactivity scores (4.95%). The results of a generalized linear mixed regression analysis showed that age, duration of illness, and parental overprotection were significant factors influencing abnormal EBPs in children (*p* < 0.05). A POM score threshold of 37 exhibited good sensitivity (74%) and specificity (90%) in predicting abnormal EBPs in children.

**Conclusion:**

Pediatric hematologic cancer patients under excessive parental protection are more prone to experiencing EBPs. Healthcare professionals should guide parents to reduce this excessive protection, thus mitigating the occurrence of EBPs in children.

## 1 Introduction

Blood cancer, as a common malignant tumor type in children, has emerged as a major disease posing a threat to life and health (Steliarova-Foucher et al., 2017). From 1990 to 2019, childhood leukemia in China accounted for over 50% of childhood cancers, consistently ranking highest in new cases and cancer-related deaths (Zhu et al., 2022). During the treatment process, children with blood cancer often have to undergo long-term chemotherapy, lumbar punctures, and other invasive diagnostic and therapeutic procedures, which can cause varying degrees of pain, nausea, hair loss, and other side effects and complications (Skeens et al., 2019). In the preschool period, children are typically in a critical phase of rapid emotional and cognitive development. During this period, children actively develop self-awareness, emotional awareness, and social skills, which are crucial for their overall growth (Klenberg et al., 2001). However, when confronted with various challenges, such as the side effects of hematologic tumor treatments, they may be more prone to experiencing emotional and behavioral problems (EBPs) (Willard et al., 2017). Some children may exhibit negative behaviors, such as refusal to cooperate with medical staff and difficulty in being comforted. Over time, some children may even develop poor social interaction, isolated personalities, and poor adaptability, which can have profound long-term effects on their lives (Okado et al., 2018).

Parenting style plays a significant role in the psychological and behavioral development of children (Chaplin and Mauro, 2022). The care and understanding parents provide are crucial factors for the normal psychological and behavioral development of their children. However, inappropriate parenting practices can contribute to difficulties in emotional and behavioral regulation in children (Morris et al., 2017). Parental overprotection (POP) is defined as a behavior in which parents excessively monitor and restrict their child’s activities and daily lives, encouraging dependence on parents and interfering with the child’s autonomy and emotional independence (Parker, 1981). This excessive protective behavior, relative to the child’s developmental stage, has been found to be closely associated with negative developmental outcomes, particularly the development of anxiety symptoms and emotional disorders (Fernandes et al., 2022).

Research has found that maternal overprotection is associated with increased symptoms of impulsivity and inattention during childhood in preterm children (Faleschini et al., 2020). Individuals who experience maternal overprotection during childhood are also more likely to experience peer victimization and have an increased risk of developing anxiety disorders in adulthood. Furthermore, excessive parental protection or control has been linked to a higher likelihood of depression and feelings of loneliness in adolescents with heart disease (Carey et al., 2002). Children with blood cancer, due to their prolonged exposure to chemotherapy and weakened immune function, are often perceived by parents as more vulnerable. As a result, these parents may exhibit excessively permissive and overprotective behaviors.

Parenting styles, like other human behavioral processes, are influenced by social and cultural factors. Different cultural groups exhibit variations in parenting practices, which, in turn, affect the developmental outcomes of children (Huang et al., 2017). This cultural factor becomes particularly relevant when considering the special circumstances of an ailing child. As children with illnesses face risks of various side effects and complications during treatment, it is understandable that parents may respond with overprotective behaviors. However, it is worth noting that, in China, both parents and healthcare professionals have relatively limited awareness of the phenomenon of POP, and there is a relative lack of relevant health education. Therefore, to gain a deeper understanding of the impact of overprotection on children’s emotions and behavior, it is necessary to conduct more in-depth research to clarify the influencing factors and the extent of its effect on children’s well-being. Furthermore, we emphasize the importance of encouraging healthcare professionals to actively engage in the treatment process of pediatric hematologic cancer patients and provide parents with more relevant health education and support.

Therefore, the main objectives of our study are as follows:

(i) To utilize a multi-center collaborative research approach to describe the current status of EBPs in Chinese pediatric hematologic cancer patients, as well as the phenomenon of POP; and (ii) to explore the impact of POP and demographic factors (e.g., age, gender, only-child status, and parents’ education level) on children’s EBPs.

## 2 Materials and methods

### 2.1 Study design, setting, and participants

Due to the challenging nature of treating pediatric blood diseases and malignancies, which involves multiple disciplines and medical institutions, specialized pediatric hospitals for blood diseases have been established in China to improve medical treatment capacity and management levels. This study adopted a multi-center collaborative research approach carried out by our research team, consisting of six graduate students, to conduct on-site investigations. These graduate students received comprehensive training, which covered techniques for engaging with the subjects, methods of inquiry, explaining questionnaire items to patients, using consistent guidance language, ensuring data quality and consistency, as well as standardizing data recording procedures. Upon arriving at the local hospital, we actively obtained consent from the management of three children’s hospitals. With the assistance of the head nurse of the Hematology-Oncology ward, we rigorously screened the study participants and obtained written consent from both the patients and their parents. We scheduled times reasonably, typically during the patients’ available free periods or when there was less medical treatment activity, to distribute the survey questionnaires to the subjects. These questionnaires were completed on-site by the children’s parents, and it usually took 15–20 min to complete one questionnaire. Subsequently, we collected the questionnaires and conducted accuracy checks.

Considering the feasibility of our study and the resource constraints within our research team, we employed convenience sampling and selected three hospitals for our investigation: Xinhua Hospital, Shanghai Jiao Tong University School of Medicine; Children’s Hospital, Zhejiang University School of Medicine; and Yuying Children’s Hospital, Wenzhou Medical University. These three hospitals were among the earliest in China to implement standardized leukemia treatments for children and have substantial research populations, which allowed us to access a larger sample size, facilitating a more comprehensive study. We selected the parents of children admitted to these three hospitals between August 2022 and June 2023 as the survey participants. The inclusion criteria were as follows: (1) Children diagnosed with blood tumor diseases (e.g., leukemia and lymphoma) based on pathological or cytological examinations; (2) Children aged 3–8 years at the time of registration; (3) Children with a disease course of 1 month; (4) Parents living with the children and participating in their care, being capable of normal communication, and willing to participate in this study. The exclusion criteria were as follows: (1) Children with attention deficit hyperactivity disorder or other mental illnesses (e.g., autism and depression); (2) Children with critical or unstable conditions requiring special parental care; (3) Children and their parents participating in other psychological intervention studies.

### 2.2 Variables and measurements

The researchers designed a questionnaire based on a literature review and pre-survey. The questionnaire included general information about the children and their parents, such as the child’s gender, age, personality, whether they are an only-child, and duration of illness, as well as the parents’ age, educational level, family structure, place of residence, average monthly income *per capita*, and their relationship with the child. The questionnaire also included disease-related information such as the child’s illness type and duration. Before conducting the survey, we conducted a preliminary survey at the nearby Wenzhou Medical University Maternal and Child Health Hospital using a convenience sampling method. We selected 5–10 participants for questionnaire completion. This process was instrumental in helping us identify inappropriate expressions and survey items, which were subsequently adjusted. This ensured that the final survey questionnaire was more accurate, easier to comprehend, and better aligned with the specific requirements of our study.

### 2.3 Emotional and behavioral problems

This study utilized the Strengths and Difficulties Questionnaire (SDQ) Parent Version, which is widely used in China to assess children’s EBPs (Mellor et al., 2016; Wang et al., 2019). The SDQ Parent Version consists of five dimensions: emotional symptoms, conduct problems, hyperactivity/inattention, peer relationship problems, and prosocial behavior, totaling 25 items. Each item is rated on a 3-point scale (0–2) ranging from “not true” to “certainly true,” with items 7, 11, 14, 21, and 25 reverse-scored. The SDQ Total Difficulties Score (TDS) is derived from the sum of scores from the first four dimensions, with higher scores indicating more severe difficulties, whereas a higher score on prosocial behavior indicates better child functioning. The Chinese version of the SDQ demonstrates a Cronbach’s α coefficient of 0.860.

We used the Chinese reference value to divide the total score of each dimension of the SDQ Parent Version into abnormal and normal groups (Zhou et al., 2020). The range of specific outliers was as follows: ≥5 for emotional symptoms, ≥4 for behavioral problems, ≥7 for hyperactivity/inattention, ≥4 for peer relationship problems, ≤4 for prosocial behaviors, and ≥ 17 for TDS.

### 2.4 Parental overprotection

The Parental Overprotection Measure (POM) was developed by Edwards et al. (Kennedy et al., 2009) in 2009 to measure parental overprotective behaviors. In this study, we used the Chinese version of the POM, which was introduced and revised by Gao et al. (2019) in 2019 for use with children aged 3–8 years. The revised version of the questionnaire has a Cronbach’s α coefficient of 0.840.

The Chinese version of the POM consists of five dimensions: excessive avoidance, extensive protection, overindulgence, avoidance of challenges, and hovering care. It includes 17 items that focus on specific behaviors or mental states of parents (e.g., “I keep a close eye on my child all the time”). Each item is rated on a 5-point scale (0–4) ranging from “never” to “always,” with a higher score indicating a higher degree of POP for the child.

### 2.5 Statistical methods

We used Excel 2021, SPSS 26.0, and SPSSAU22.0 analysis systems for data entry and statistical analysis. We used the P–P chart and the Kolmogorov–Smirnov test to verify whether the continuous variables fit a normal distribution. The measurement data were of a non-normal distribution and are expressed as medians (interquartile distances) [M(P25, P75)]. The counting data are expressed as numbers of cases and percentages (%). We used generalized linear mixed regression analysis to explore the influencing factors of EBPs in children with blood tumors.

### 2.6 Research ethics approval

We obtained approval from the Ethics Committee of Yuying Children’s Hospital, the Second Affiliated Hospital of Wenzhou Medical University (2022-K- 164-02), to conduct this study. After obtaining consent from the guardians of children with blood cancer, we entered each ward accompanied by nursing staff to conduct the investigations and collect relevant information about the children. To ensure the confidentiality of the data, the children’s names were replaced with numerical codes and only authorized personnel were permitted access to the list.

## 3 Results

### 3.1 Participants

This study included a total of 212 families who participated in the questionnaire survey. There were 202 valid questionnaires, resulting in a questionnaire response rate of 95%. Regarding the types of blood cancers in children, leukemia accounted for 177 cases (87.6%), lymphoma accounted for 17 cases (8.4%), and neuroblastoma accounted for eight cases (4%). The respondents included 176 mothers (87. 1%) and 26 fathers (12.9%). Table 1 provides further details of the general characteristics of the study participants.

**Table 1:** Participant characteristics (*N* = 202).

Category	*n*	%	Category	*n*	%
Age of child (years)			Educational level of the parents		
3–4	70	34.7	High school or below	90	44.6
5–6	91	45.0	Junior college	67	33.2
7–8	41	20.3	Bachelor’s degree or higher	45	22.3
Gender of child			Area of residence		
Male	130	64.4	Village	61	30.2
Female	72	35.6	Town	61	30.2
Number of siblings			City	80	39.6
0	74	36.6	Family type		
≥1	128	63.4	Core family	121	59.9
Temperament of the child			Stem family	72	35.6
Extroverted	136	67.3	Other families	9	4.5
Introverted	66	32.7	Monthly household income (in CNF)		
Time since diagnosis (months)			≤3,000	43	21.3
1–3	53	26.2	3,001–5,000	73	36.1
4–6	57	28.2	5,001–8,000	73	36.1
7–11	37	18.3	>8,000	13	6.44
≥12	55	27.2			

### 3.2 Child emotional and behavioral problems: SDQ scores

According to the scoring criteria of the questionnaires, Table 2 presents the parents’ SDG scores and the number of children with EBPs (i.e., detection rate). The specific results were as follows: 56 individuals (27.72%) had abnormal emotional symptoms, 29 (14.36%) had abnormal conduct problems, 10 (4.95%) had abnormal hyperactivity, 53 (26.24%) had abnormal peer relationship problems, 52 (25.74%) had abnormal prosocial behavior, and 11 (13.86%) had an abnormal TDS.

**Table 2:** SDQ scores.

Scale dimension	Scale score median (IQR)	Abnormal number (n)*	Anomaly rate (%)*
Emotional symptoms	3 (2.00,5.00)	56	27.72
Conduct problems	2 (2.00,3.00)	29	14.36
Hyperactivity/ inattention	3 (2.00,4.00)	10	4.95
Peer problems	2 (1.75,4.00)	53	26.24
Prosocial behavior	5.50 (4.00,7.00)	52	25.74
TDS	11 (9.00, 14.00)	28	13.86

Frequencies presented are row percentages (the number and the % of individuals with emotional and behavioral problems in each dimension of the scale).

IQR, interquartile range; *n*, number; TDS, total difficulty score.

### 3.3 Parental overprotection: POM questionnaire scores

Table 3 shows the parents’ scores on each dimension and the total score of the Chinese version of the POM questionnaire. The median score for overall POP was 37 (IQR = 32.75–44). The median scores for each dimension were as follows: excessive avoidance = 8 (IQR = 6–9), extensive protection = 11 (IQR = 10–13), excessive indulgence = 6 (IQR = 4–7), avoidance of challenges = 6 (IQR = 5–8), and overprotection = 7 (IQR = 5–8).

**Table 3:** POM questionnaire scores.

Scale dimension	Scale score median (IQR)
Excessive avoidance	8 (6.00,9.00)
Excessive protection	11 (10.00,13.00)
Excessive indulgence	6 (4.00,7.00)
Avoidance of challenges	6 (5.00,8.00)
Close monitoring	7 (5.00,8.00)
Total POP score	37 (32.75,44.00)

IQR, interquartile range; n, number; TDS, total difficulty score; POP, parental overprotection.

### 3.4 General information of children and results of unifactor analysis of abnormal emotional behavior

As shown in Table 4, there were significant differences (*p* < 0.05) in the detection rates of EBPs among children based on their age (OR = 0.216, 95% CI = 0.132–0.356), temperament (OR = 3.301, 95% CI = 1.695–6.43), disease duration (OR = 0.277, 95% CI = 0.196–0.392), parents’ education level (OR = 0.19, 95% CI = 0.118–0.306), long-term residential location of the family (OR = 0.288, 95% CI = 0.190–0.437), and monthly income *per capita* of the family (OR = 0.358, 95% CI = 0.242–0.53).

**Table 4:** Results of univariate analysis of children’s general data and abnormal EBPs.

Variable	Whether EBPs were abnormal n (%)*		OR (95% CI)	Value of *p*
	No	Yes		
Age of child (years)				
3–4	8 (9.76)	62 (51.67)		
5–6	44 (53.66)	47 (39.17)	0.216 (0.132–0.356)	**<0.001** ^a^
7–8	30 (36.59)	11 (9.17)		
Gender of child				
Male	50 (60.98)	80 (66.67)	0.781 (0.436–1.401)	0.407
Female	32 (39.02)	40 (33.33)		
Number of siblings				
0	27 (32.93)	47 (39.17)	0.762 (0.423–1.374)	0.367
≥1	55 (67.07)	73 (60.83)		
Temperament of the child				
Extroverted	67 (81.71)	69 (57.50)	3.301 (1.695–6.43)	**<0.001** ^a^
Introverted	15 (18.29)	51 (42.50)		
Time since diagnosis (months)				
1–3	3 (3.66)	50 (41.67)		
4–6	16 (19.51)	41 (34. 17)		
7–11	19 (23. 17)	18 (15.00)	0.277 (0.196–0.392)	**<0.001** ^a^
≥12	44 (53.66)	11 (9. 17)		
Educational level of the parents				
High school or below	8 (9.76)	82 (68.33)		
Junior college	42 (51.22)	25 (20.83)	0.19 (0.118–0.306)	**<0.001** ^a^
Bachelor’s degree or higher	32 (39.02)	13 (10.83)		
Area of residence				
Village	7 (8.54)	54 (45.00)		
Town	24 (29.27)	37 (30.83)	0.288(0.190 ~ 0.437)	**<0.001** ^a^
City	51 (62.20)	29 (24.17)		
Family type				
Core family	50 (60.98)	71 (59.17)		
Stem family	26 (31.71)	46 (38.33)	0.915 (0.564–1.482)	0.717
Other families	6 (7.32)	3 (2.50)		
Monthly household income (in CNF)				
≤3,000	7 (8.54)	36 (30.00)		
3,001–5,000	24 (29.27)	49 (40.83)		
5,001–8,000	39 (47.56)	34 (28.33)	0.358 (0.242–0.53)	**<0.001** ^a^
>8,000	12 (14.63)	1 (0.83)		

Bold values denote statistical significance at the *p* < 0.05 level.

*Frequencies presented are row percentages (the number and the % of individuals with emotional and behavioral problems in groups divided by different factors).

^a^Chi-square test.

EBPs, emotional and behavioral problems.

### 3.5 Results of multivariate analysis of children’s emotional and behavioral problems

Children aged 3–4 years had a higher likelihood of experiencing EBPs compared to those aged 7–8 years (OR = 0.216, 95% CI = 0.132–0.356). Additionally, shorter disease duration was associated with a higher likelihood of EBPs in children (OR = 0.277, 95% CI = 0.196–0.392). Moreover, children with parents who had lower education levels were more likely to have EBPs (OR = 0.19, 95% CI = 0.118–0.306). Furthermore, higher POM scores were associated with an increased likelihood of EBPs in children (Table 5).

**Table 5:** Results of multivariate analysis of children’s emotional behavior problems.

Variable OR (95% CI)		Value of *p*	Variable OR (95% CI)		Value of *p*
Age of child (years)			Educational level of the parents		
3–4	0.071 (0.015–0.324)	**0.001** ^a^	High school or below	0.241 (0.062–0.944)	**0.041** ^a^
5–6	0.338 (0.094–1.218)	0.097	Junior college	1.002 (0.308–3.26)	0.998
7–8			Bachelor’s degree or higher		
Time since diagnosis (months)			Monthly household income (in CNF)		
1–3	0.091 (0.014–0.572)	**0.011** ^a^	≤3,000	0.256 (0.016–4. 106)	0.334
4–6	0. 169 (0.053–0.537)	**0.003** ^a^	3,001–5,000	0.197 (0.016–2.429)	0.204
7–12	0.383 (0.115–1.271)	0.116	5,001–8,000	0.19 (0.015–2.339)	0.193
>13			>8,000		
Temperament of the child			Area of residence		
Extroverted	0.865 (0.266–2.815)	0.809	Village	0.447 (0.106–1.892)	0.273
Introverted			Town	0.668 (0.226–1.981)	0.466
POM	0.046 (0.007–0.306)	**0.002** ^a^	City		

Bold values denote statistical significance at the *p* < 0.05 level.

^a^Generalized linear mixed regression model.

## 4 Discussion

In this study, the children’s TDSs were significantly higher than those of children in elementary and junior high schools in Japan and China (Takeshima et al., 2021; Zhou et al., 2022). This result could be attributed to the fact that hematologic malignancies involve a series of stressors that may trigger negative psychological reactions (Sandra et al., 2022), resulting in reported scores that are lower than those of healthy children. Additionally, the majority of participants in this study were children of preschool age, who are younger than those in elementary or junior high school. Consistent with the findings of Sleurs et al. (2021), our results indicated that age is an important influencing factor for EBPs in children. This correlation may be because younger children have fewer coping strategies available to them during the disease treatment process, whereas children aged ≥6 years can employ strategies (e.g., attention diversion and cognitive self- guidance) that help them adapt to the treatment environment and reduce emotional distress (Tremolada et al., 2022). Manijeh et al.’s study indicated that younger children show higher sensitivity to pain (Firoozi and Rostami, 2012). Moreover, preschool-age children are more prone to experiencing neurobehavioral side effects when undergoing treatment with corticosteroids; this tendency may be attributed to the incomplete development of their brain regulatory systems, which makes them more susceptible to the adverse effects of corticosteroids (Mrakotsky et al., 2011; Samsel and Muriel, 2017).

Among our study population, 56 children (27.72%) had abnormal emotional symptoms, 53 (26.24%) had abnormal peer problems, and 52 (25.74%) had abnormal prosocial behavior. These rates were much higher compared to the findings of Mader et al. (2022) in their study on pediatric cancer survivors. This difference may be attributed to the fact that this survey mainly focused on children undergoing treatment, who seem to experience more emotional distress compared to children who have completed treatment. During the initial diagnosis, children may face new awareness and acceptance of their illness, as well as the initiation of treatments such as chemotherapy and surgery, which can trigger intense emotional and stress reactions (Schilstra et al., 2022). Throughout the treatment process and post-treatment, many children experience emotional distress (Kunin-Batson et al., 2016). However, as the treatment progresses and the child adapts over time, the level of distress tends to decrease, suggesting that children gradually adjust to the treatment process and their condition, leading to a reduction in EBPs (Okado et al., 2016; Mant et al., 2019).

This study found that POP is an important factor influencing the EBPs of children with blood tumors. This finding is consistent with the results of Kim et al. (2015), who found a positive correlation between POP and negative self-perception and hostile emotions in children with leukemia in Germany. In terms of the children’s perspective, Ernst et al. (2019) found that those with leukemia and lymphoma are more likely to recall perceiving POP as a child. Additionally, in a study by Tillery, children who perceived POP tended to experience higher levels of distress (Tillery et al., 2014).

In Chinese parenting culture, parents typically prioritize the protection of their children and tend to limit their free playtime due to concerns about potential injuries or infections. This tendency may be influenced by the emphasis on family bonds and the cultivation of close relationships within Chinese families (Harkness, 2023). Furthermore, POP can have long-term effects on children’s adaptability (Fedele et al., 2011). Children may lack opportunities to interact with peers and develop social skills, which can ultimately impact their social adaptation.

The results of this study also indicate that children of parents with lower education levels are more likely to experience EBPs. This result could be because parents with higher education levels may possess more knowledge about child-rearing, which reduces overprotective behaviors (De La Maza et al., 2020). Parents with higher education levels are generally better equipped to handle emotional issues concerning their children (Alicandro et al., 2020). Additionally, parental distress and negative emotions are associated with a higher prevalence of EBPs in children (Van Der Geest et al., 2014). Parents with higher education levels are more likely to benefit from support from friends and others, enabling them to better cope with life’s stressors (Salvador et al., 2019).

In China, insufficient attention has been given to researching POP. This study, which was conducted in a specific Chinese context, utilized a multicenter cross-sectional survey, thereby filling a knowledge gap in this field. The limitations of this study include its reliance on parental completion of the survey questionnaire, which may not fully reflect the children’s own experiences. Additionally, we did not consider the perspectives of intergenerational caregivers. Research has indicated that the caregiving styles of grandparents are also related to EBPs in children (Li et al., 2019). To gain a more comprehensive understanding of the factors influencing EBPs in children with hematologic tumors, future studies could incorporate the viewpoints of intergenerational caregivers and explore the children’s experiences and perspectives from different angles and dimensions. Another significant limitation of our study is the absence of a healthy control group. The inability to make comparisons with a healthy control group could potentially affect the depth of our understanding of the relationship between POP and the psychological and behavioral development of children. For future research, it is advisable to consider including a healthy control group to yield more reliable conclusions and recommendations.

## 5 Conclusion

In this study, we observed a relatively higher incidence of EBPs in children with blood cancer, especially in younger children and those recently diagnosed. Simultaneously, we also noted that this issue was more prominent in children whose parents exhibited higher levels of protection. Although parental support and care are crucial for these children, it is essential to avoid excessive protection. Therefore, we encourage healthcare professionals to provide educational and supportive programs targeting excessive protection. These programs, which should encompass comprehensive health education and parental guidance, can help alleviate parents’ anxiety and prevent excessive interference while reducing their concerns. These measures will motivate parents to seek a more balanced approach to their children’s disease treatment, thereby promoting their psychological well- being. By doing so, we can collectively mitigate potential long-term effects of disease treatment in these children and enhance the psychological well-being of children with blood cancer.

## Data availability statement

The data analyzed in this study is subject to the following licenses/restrictions: The raw data supporting the conclusions of this article will be made available by the authors, without undue reservation. Requests to access these datasets should be directed to CZ, sallyzcm@wmu.edu.cn.

## Ethics statement

The studies involving humans were approved by Medical Ethics Committee of the Second Affiliated Hospital of Wenzhou Medical University, Yuying Children’s Hospital of Wenzhou Medical University AF/SW-01-3.0. The studies were conducted in accordance with the local legislation and institutional requirements. Written informed consent for participation in this study was provided by the participants’ legal guardians/next of kin.

## Author contributions

YY: Conceptualization, Data curation, Investigation, Methodology, Software, Writing – original draft, Writing – review & editing. XZ: Conceptualization, Methodology, Project administration, Supervision, Writing – review & editing. WX: Investigation, Methodology, Software, Writing – review & editing. YH: Data curation, Investigation, Software, Writing – review & editing. XW: Data curation, Investigation, Software, Writing – review & editing. WH: Formal analysis, Investigation, Validation, Writing – review & editing. RW: Formal analysis, Project administration, Resources, Writing – review & editing, Investigation. XY: Writing – review & editing, Investigation, Project administration, Validation. CZ: Conceptualization, Resources, Supervision, Writing – original draft.
